# Immune Dysregulation and the Increased Risk of Complications and Mortality Following Respiratory Tract Infections in Adults With Down Syndrome

**DOI:** 10.3389/fimmu.2021.621440

**Published:** 2021-06-25

**Authors:** Tomer Illouz, Arya Biragyn, Maria Florencia Iulita, Lisi Flores-Aguilar, Mara Dierssen, Ilario De Toma, Stylianos E. Antonarakis, Eugene Yu, Yann Herault, Marie-Claude Potier, Alexandra Botté, Randall Roper, Benjamin Sredni, Jacqueline London, William Mobley, Andre Strydom, Eitan Okun

**Affiliations:** ^1^ The Leslie and Susan Gonda Multidisciplinary Brain Research Center, Bar-Ilan University, Ramat Gan, Israel; ^2^ The Paul Feder Laboratory on Alzheimer’s Disease Research, Bar-Ilan University, Ramat Gan, Israel; ^3^ Laboratory of Molecular Biology and Immunology, National Institute on Aging, National Institute of Health, Baltimore, MD, United States; ^4^ Sant Pau Memory Unit, Department of Neurology, Hospital de la Santa Creu i Sant Pau, Barcelona, Spain; ^5^ Biomedical Research Institute Sant Pau, Universitat Autònoma de Barcelona, Barcelona, Spain; ^6^ Center of Biomedical Investigation Network for Neurodegenerative Diseases (CIBERNED), Madrid, Spain; ^7^ Department of Anatomy and Cell Biology, McGill University, Montreal, QC, Canada; ^8^ Center for Genomic Regulation, The Barcelona Institute for Science and Technology, Barcelona, Spain; ^9^ University Pompeu Fabra, Barcelona, Spain; ^10^ Biomedical Research Networking Center for Rare Diseases (CIBERER), Barcelona, Spain; ^11^ Department of Genetic Medicine and Development, University of Geneva, Geneva, Switzerland; ^12^ Medigenome, Swiss Institute of Genomic Medicine, Geneva, Switzerland; ^13^ iGE3 Institute of Genetics and Genomics of Geneva, Geneva, Switzerland; ^14^ The Children’s Guild Foundation Down Syndrome Research Program, Genetics and Genomics Program and Department of Cancer Genetics and Genomics, Roswell Park Comprehensive Cancer Center, Buffalo, NY, United States; ^15^ Genetics, Genomics and Bioinformatics Program, State University of New York at Buffalo, Buffalo, NY, United States; ^16^ Université de Strasbourg, CNRS, INSERM, Institut de Génétique Biologie Moléculaire et Cellulaire, IGBMC - UMR 7104 - Inserm U1258, Illkirch, France; ^17^ Paris Brain Institute (ICM), CNRS UMR7225, INSERM U1127, Sorbonne Université, Hôpital de la Pitié-Salpêtrière, Paris, France; ^18^ Department of Biology, Indiana University-Purdue University Indianapolis, Indianapolis, IN, United States; ^19^ The Mina and Everard Goodman Faculty of Life Sciences, Bar-Ilan University, Ramat Gan, Israel; ^20^ Université de Paris, BFA, UMR 8251, CNRS, Paris, France; ^21^ Department of Neurosciences, University of California, San Diego, San Diego, CA, United States; ^22^ Department of Forensic and Neurodevelopmental Sciences, Institute of Psychiatry Psychology and Neuroscience, King’s College London, London, United Kingdom; ^23^ South London and Maudsley NHS Foundation Trust, London, United Kingdom

**Keywords:** Down syndrome, immune dysregulation, hospitalization, respiratory tract infections, interferon, COVID-19

## Abstract

The risk of severe outcomes following respiratory tract infections is significantly increased in individuals over 60 years, especially in those with chronic medical conditions, i.e., hypertension, diabetes, cardiovascular disease, dementia, chronic respiratory disease, and cancer. Down Syndrome (DS), the most prevalent intellectual disability, is caused by trisomy-21 in ~1:750 live births worldwide. Over the past few decades, a substantial body of evidence has accumulated, pointing at the occurrence of alterations, impairments, and subsequently dysfunction of the various components of the immune system in individuals with DS. This associates with increased vulnerability to respiratory tract infections in this population, such as the influenza virus, respiratory syncytial virus, SARS-CoV-2 (COVID-19), and bacterial pneumonias. To emphasize this link, here we comprehensively review the immunobiology of DS and its contribution to higher susceptibility to severe illness and mortality from respiratory tract infections.

## Overview

**Figure 1:**
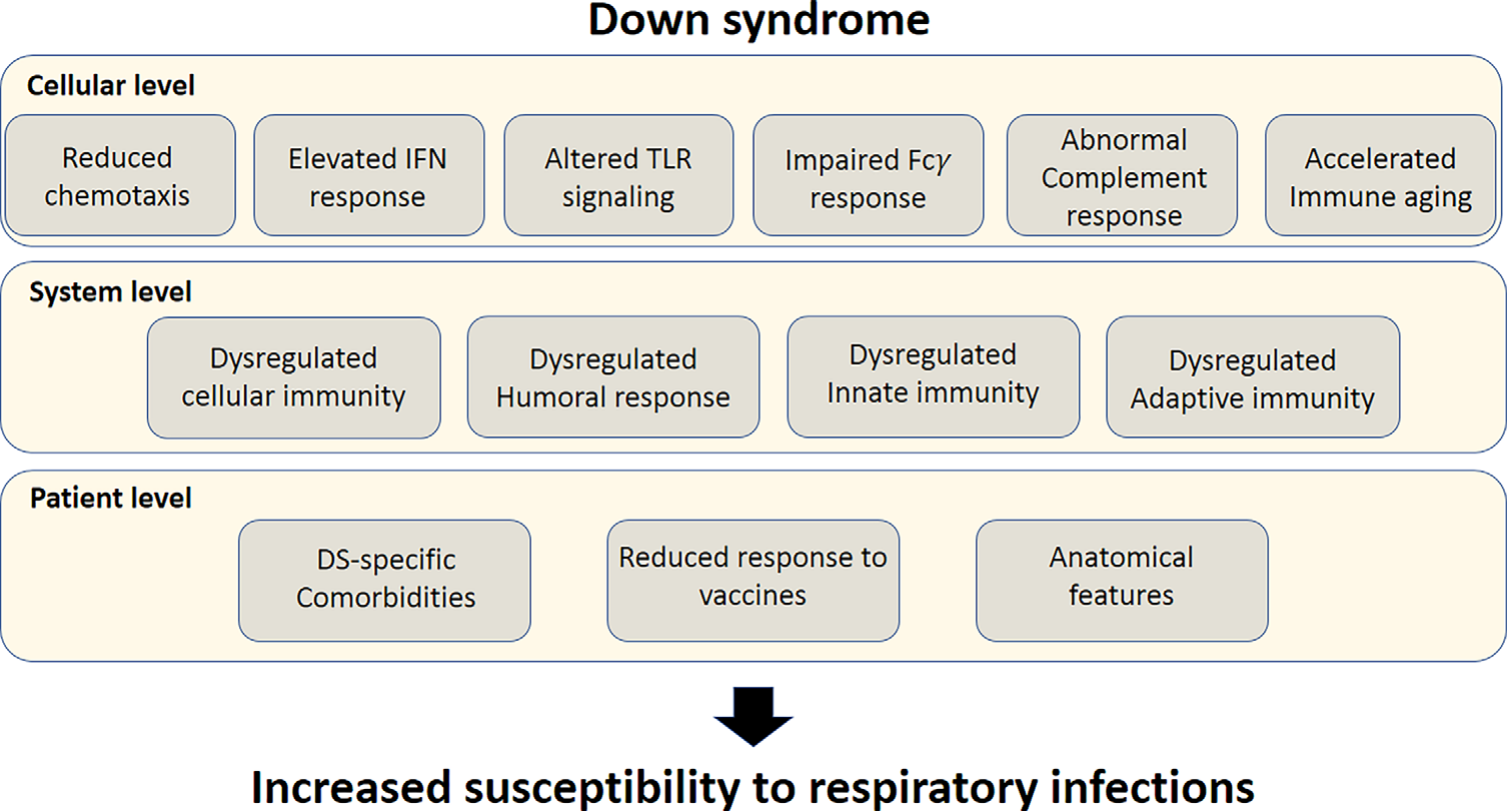
Susceptibility factors to severe response to respiratory tract infections in Down syndrome. Top bar; Cellular-level susceptibility factors. Middle bar; System-level susceptibility factors. Lower bar; patient-level susceptibility factors. All these factors culminate in increased susceptibility of individuals with DS to respiratory tract infections.

**Figure 2:**
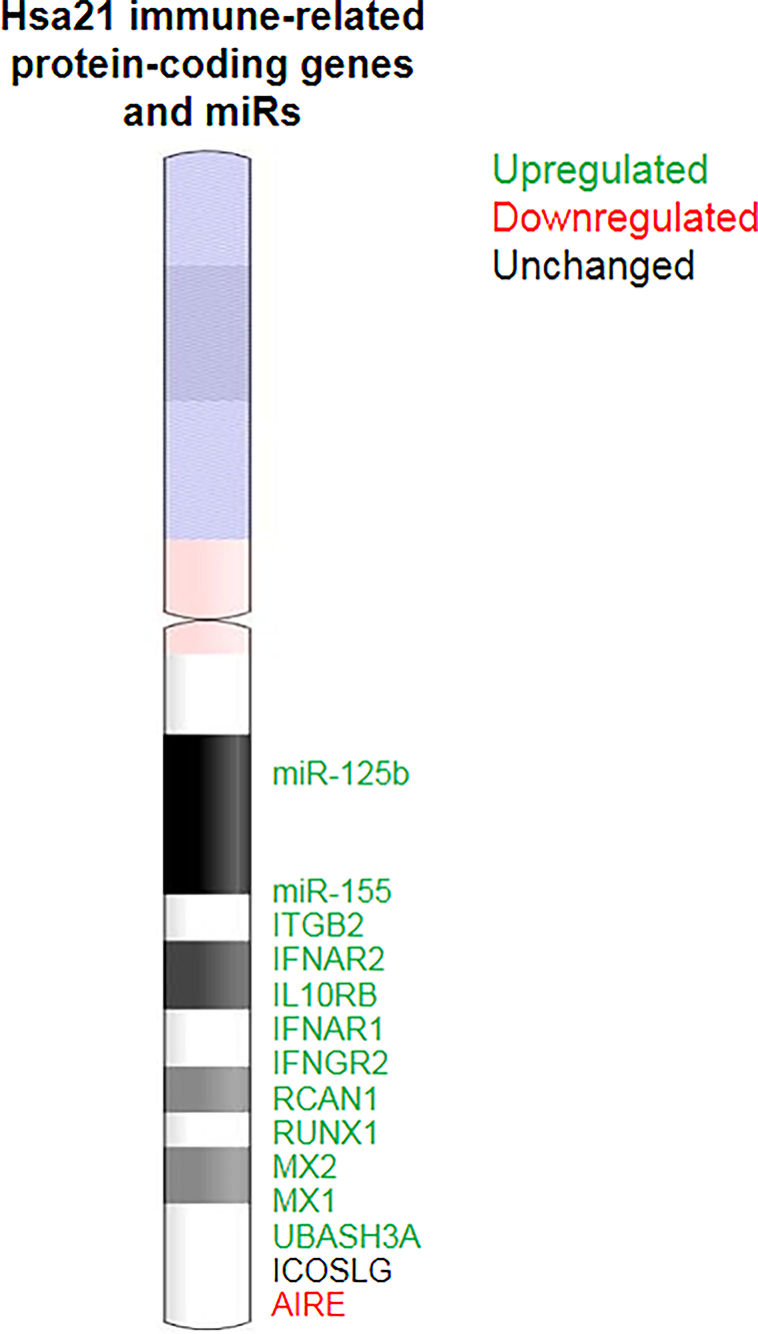
Immune-related genes in Chr21. Alterations in the expression of Chr21 immune-related genes and miRs may directly contribute to immune dysregulation in DS.

Individuals with Down syndrome (DS) exhibit a higher risk of developing severe responses to infectious diseases compared to the general population (1–9). Chromosome 21 (Chr21), which is triplicated in DS, harbors several essential immune-related genes, such as four out of the six subunits of IFN receptors (10), β-2 integrin (ITGB2) (11), ubiquitin associated and SH3 domain containing A (UBASH3A) (12), autoimmune regulator (AIRE) (13), and more (**Figures 1**, **2**). As a result, the immune system of individuals with DS is often altered or dysregulated. Immune dysregulation can be caused by mutations in key immune regulatory genes, or increased gene dosage, as in the case of DS (11, 14). Accordingly, the adaptive and innate immune responses in individuals with DS are aberrant at multiple levels, including cellular anomalies (15–20), reduced humoral response (21), elevated interferon (IFN) signaling (10, 22), and altered toll-like receptor (TLR) signaling (23), along with accelerated aging of the immune system (24) (**Figure 1**).

At the individual level, the immune dysregulation observed in DS is associated with blunted response to vaccines (21, 25) and increased risk of infections, more prominently in the respiratory tract (8, 9). Moreover, the risk of respiratory infections may be exacerbated by unique anatomical airway features of individuals with DS (26) (**Figure 1**).

### Genetics of DS

DS is the most common genetic disorder causing a variable degree of intellectual disability (ID). It was first clinically described in 1866 by Langdon Down (27). Almost a century later, in 1959, trisomy for the small acrocentric Chr21 was identified as the genomic cause of this syndrome (28). The DS genome usually contains an entire supernumerary Chr21, while translocation of the entire Chr21 to another chromosome accounts for ~ 5% of all DS cases (29, 30). In rare cases, only a portion of Chr21 is triplicated, resulting in a partial trisomy (31, 32). The majority of individuals with DS (90–95%) are trisomic for Chr21 in all of their cells, whereas a small proportion are mosaic (33).

Chr21 is the smallest human chromosome (~1.46% of the genome). The sequence of its long arm was published in 2000 (34). Trisomy-21 mostly results from a chromosome segregation error in maternal meiosis I or II; the remainder are due to errors in paternal meiosis I or II, as well as mitotic postzygotic errors (35). Maternal age is a major risk factor for DS since aging mothers have a higher chance of a DS pregnancy (36), although the exact mechanism of this is unknown.

Chr21 contains approximately 233 protein-coding genes (GRCh38.p13, Genecode database), 423 non-protein-coding genes, and numerous other functional genomic elements (29) that may be of importance for the phenotypic variability of DS. In this respect, DS is considered a disorder of altered gene expression (29). The consequences of gene dosage imbalance, including in immune related genes (**Figure 2**), is widespread and include immune dysregulation (22).

### Worldwide Prevalence of DS

DS occurs in all populations, with small differences in the incidence rates in various countries or population groups influenced mainly by maternal age at conception (36). On average, the number of newborns with DS is ~12.8 per 10,000 births (~1 in 780 newborns), with more males with DS than females across most countries (37, 38). This number is strongly influenced by the practice of prenatal detection and subsequent elective termination of pregnancies. In several countries, however, increasing maternal age counterbalances the impact of prenatal diagnosis and results in a stable prevalence (37).

### Changes in Lifespan of Individuals With DS

The last two generations have seen marked increase in the lifespan of individuals with DS. In the 1940s, the average life expectancy for people with DS was 12 years (39). Today, individuals with DS have a life expectancy of 60 years on average (40), which is mostly linked to advances in medical care. The main causes of death in adults with DS are Alzheimer’s disease (AD), congenital heart defects, and pneumonia, in contrast to solid tumors and ischemic heart disease, which dominate in the general population (41–43). Increased survival is in part attributable to early surgical interventions of congenital heart malformations. Nevertheless, the risk for premature mortality is high in DS, mainly because of respiratory infections and leukemia (44). As a result of their relatively longer life expectancy, a greater number of individuals with DS now suffer from aging-related pathologies such as dementia, skin and hair changes, early onset menopause, visual and hearing impairments, adult-onset seizure disorder, thyroid dysfunction, diabetes, obesity, sleep apnea, and musculoskeletal problems (45).

## Infectious Diseases in Adults With DS

Despite the increase in life expectancy of individuals with DS, children with DS remain at higher risk of neonatal and infant mortality compared with children without DS (46). Children with DS have a high incidence of ear, nose, and throat infections (47) and respiratory tract infections (RI), which represent a leading cause of premature mortality (48). A special vulnerability to infections affects individuals with DS after the age of 50 years, especially with certain co-morbidities, such as seizures (49). Individuals with DS suffer from a more severe illness following respiratory tract infections, primarily with influenza virus (8), respiratory syncytial virus (RSV), parainfluenza virus (1), SARS-CoV-2 (9, 50), and Streptococcus pneumoniae (51). Skin (52) and periodontal tissue infections (53) are also prevalent in individuals with DS.

### Respiratory Tract Infections

#### Respiratory Syncytial Virus (RSV)

RSV is a double-stranded RNA (dsRNA) virus and the primary cause of severe lower respiratory tract infections (LRI) in young children (<5 years) (3, 8). Acute LRI is characterized by cough and/or respiratory distress and requires hospitalization in 0.5–2% of pediatric cases. Children with DS are at greater risk for RSV infections (3, 5), especially for those with chronic lung disease or congenital heart disease. Almost every child with DS is infected with RSV by the age of 2 years, and nearly half develop acute LRI (54). A meta-analysis conducted by Beckhaus and Castro-Rodriguez revealed that the length of hospitalization following RSV infection is higher in children with DS (mean difference: 4.73 days). In addition, patients with DS are more likely to require oxygen support (odds ratio: 6.53), ICU admission (odds ratio: 2.56), and mechanical ventilation (odds ratio: 4.56). Mortality rate is also higher in children with DS than in children without DS (odds ratio: 9.4) (3).

Palivizumab, a humanized monoclonal antibody and the only preventative measure to RSV, is indicated for young children (<24m) with high risk (e.g., prematurity, congenital heart disease, and chronic lung disease).

To date, the American Academy of Pediatrics does not recommend the routine use of palivizumab in patients with DS without additional risk factors (3). In Japan, however, since 2013, children with DS can receive insurance-covered palivizumab even when they lack the standard indication and other medical problems. This expanded palivizumab prophylaxis program was reported to decrease RSV-related hospital admissions but was associated with neither lower RSV infection nor lower mortality rates (55). A study analyzing data from before and after the introduction of the universal palivizumab prophylaxis program for children with DS in Japan reported no reduction in RSV-related hospitalization in this population after 2013 (56), suggesting that the protective effect of palivizumab in children with DS that exhibit no additional risk factors is minor.

#### Influenza Virus

Following the outbreak of the H1NI 2009 pandemic in Mexico, a study found a 16-fold increase in hospitalization, an 8-fold increase in endotracheal intubation, and more than 300-fold increase in death in individuals with DS than in the general population (8). Moreover, patients with DS were younger compared with affected individuals in the general population (15.2 *vs*. 41.6 years, mean age, respectively). As a result, it was suggested that individuals with DS should be vaccinated against seasonal influenza and the H1N1 strain (8). A study that examined antibody production following the influenza A/H1N1 vaccine in 48 children with DS found that only 27% of the children reached an antibody level that predicts a 50% clinical protection rate (57).

#### SARS-CoV-2

The SARS-CoV-2 outbreak, which causes coronavirus disease 2019 (COVID-19), is particularly life-threatening to individuals with DS due to preexisting co-morbidities, health and housing conditions, immune dysfunction, and premature aging (9, 50). A study conducted during the pandemic using multivariate logistic regression on the ‘Leumit Healthcare Services’ database in Israel reported a significant association between DS and the likelihood of being tested positive for SARS-CoV-2 (adjusted odds ratio 1.64). Either dysregulation of the immune system, co-morbidities, or sociological factors such as housing conditions can contribute to this predisposition. Additionally, the mean age of individuals with DS who tested positive was lower compared to individuals without DS who tested positive (18.47, 31.43 years, respectively) (50).

In the most comprehensive study to date, Huls and colleagues examined the vulnerability of individuals with DS to severe COVID-19 in two large cohorts: an international cohort from the Trisomy-21 research society (T21RS) survey, and the UK ISARIC4C survey. The investigators reported that the most prevalent symptoms of COVID-19 in individuals with DS were fever, cough, and shortness of breath; similar to those reported in general population. Altered consciousness or confusion were more common in hospitalized individuals with DS compared with hospitalized individuals without DS. However, joint pain, muscle aches, and vomiting/nausea were less frequent in DS (9). 60% of DS cases from the T21RS survey, reported by clinicians, developed medical complications due to COVID-19, which correlated with higher mortality rates. Most prevalently, these complications were viral pneumonia and acute respiratory stress syndrome. These complications increased with age, from 41% of patients at age 0-19 to 65% at ages 20-39 and 69% at age 40 and above. A retrospective study that was conducted in New York and included 7246 patients hospitalized with COVID-19, 12 of them with DS, found that these patients exhibit a more severe disease than controls, particularly an increased incidence of sepsis and need of mechanical ventilation (58). In line with these reports, Clift and coworkers analyzed patient-level data of 8.26 million adults (aged >19 years), collected from January to June 2020 in the U.K., to evaluate whether DS is a risk factor for hospitalization and mortality from COVID-19. Indeed, the hospitalization hazard ratio due to COVID-19 for people with DS is 4.94, compared with people without DS (59).

The rate of COVID-19-associated mortality is also higher in adults with DS than in the general population. Among hospitalized individuals in the ISARIC4C survey, mortality has increased in DS from age 40, compared to 60 in the general population. Mortality rates under the age of 40 were low, however, hospitalization rate of individuals with DS was higher than in the general population (7%, 3%, respectively). By comparing the T21RS data with the ISARIC4 data, the researchers were able to report that individuals with DS hospitalized with COVID-19 were ~3 times more likely to die than individuals without DS, assuming the same age, gender, and ethnicity. Overall, the mortality rate after hospitalization was 13% in non-DS patients from the ISARIC4 survey, 40% in DS patients from the ISARIC4 survey, and 48% in DS patients from the T21RS survey. Before the age of 40, the mortality rate after hospitalization was 3% in non-DS patients from the ISARIC4 survey, 12% in DS patients from the ISARIC4 survey, and 12.5% in DS patients from the T21RS survey. After the age of 40, the mortality rate among hospitalized cases was 17% in non-DS patients from the ISARIC4 survey, 49% in DS patients from the ISARIC4 survey, and 55.5% in DS patients from the T21RS survey (9). In a cohort study of 8 million adults, Clift and colleagues reported that COVID-19 accounted for 39.7% of deaths among individuals with DS, and for 20.3% of deaths among individuals without DS. Adjusting for age and sex, the researchers found that the mortality hazard ratio of COVID-19 for individuals with DS is 24.94 compared with individuals without DS. Following further adjustments –ethnicity, BMI, care home residency, and comorbidities– the mortality hazard ratio was 10.39. Interestingly, this study did not find evidence for interaction between DS and age or BMI that was associated with higher hazard ratio (59).

As in the general population, age is a stronger risk factor for severe COVID-19 illness in individuals with DS. Obesity, diabetes, and congenital heart diseases, all DS-co-morbidities, along with male gender, are additional risk factors for hospitalizations with COVID-19. Risk factors for mortality were male gender and AD/dementia (9). It is therefore well evident that individuals with DS are at higher risk of SARS-CoV-2 infection (50), COVID-19-related complications (58), and mortality (9, 59).

### Infectious Diseases in DS: Hospitalization Rate and Length

Children with DS may have a greater risk of admission to a hospital and are more likely to have an extended stay and require intensive care support upon respiratory tract infections (23). However, studies have only recently begun to focus on the adult DS population with respect to their rate of infections, and the number and length of hospitalizations. In general, hospitalization rate is higher in adults with DS compared with the general population due to pneumonia or aspiration (60–62). Tenenbaum and colleagues analyzed 297 hospitalizations of 120 adults with DS (aged 18-73 years in medical centers in Israel between 1988-2007) and compared these data with a control population. The number of hospitalizations recorded for individuals with DS was twice that of individuals without DS. The average hospitalization length was more than 1.5-fold (8.1 *vs*. 4.8 days), despite no difference in the mean age of the hospitalized people (39.1 *vs*. 39.5, years). More than a fourth of the hospitalizations were caused by infectious diseases.

As noted, DS is associated with increased susceptibility infections (17, 21, 25, 63–65). Within the DS population, young children and the elderly appear to mostly suffer from respiratory infections (41, 66), and at higher rates than in the general population (67). This is associated with worse outcomes, higher hospitalization rates, more extended hospital stays, and higher mortality rates.

## Interplay Between Immune Dysregulation and Infectious Diseases in DS

**Figure 3:**
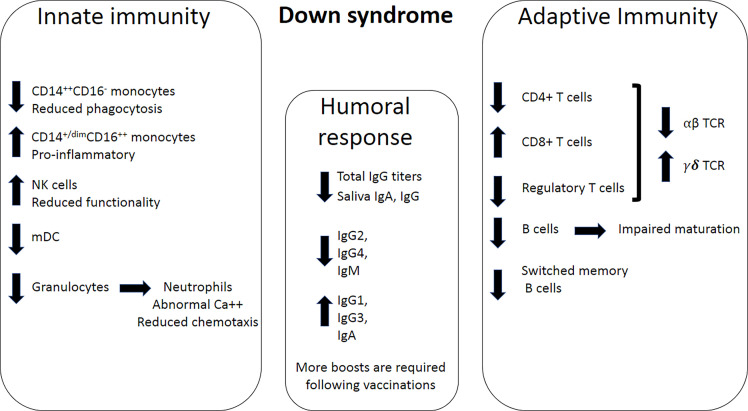
Immune dysregulation in DS. Left panel; Innate immunity impairments in DS include a decrease in CD14^++^CD16^-^ monocytes, an increase in CD14^+^CD16^+^ monocytes, increase in NK cells, a decrease in myeloid dendritic cells (mDC), and a decrease in granulocytes. Neutrophils exhibit dysregulated Ca^++^ homeostasis and reduced chemotactic ability, which results in impaired functionality. Middle panel; humoral response dysregulation includes a decrease in total IgG, as well as IgG and IgA levels in the saliva. Specifically, of IgG2, IgG4, and IgM production levels decrease, while production levels of IgG1, IgG3 and IgA increase. Right panel; Adaptive immunity impairments include a decrease in the numbers of CD4^+^ T cells, an increase in the numbers of CD8^+^ cells, and a decrease in the number of T regulatory cells. Overall, fewer T cells in DS express the αβ subunits of the T-cell receptor (TCR), and more T cells express the *γ*δ subunits of the TCR. The numbers of B cells and switched memory B cells also decrease in DS.

Multiple immunological impairments are present in DS, including dysfunction of the cellular and humoral responses, altered phagocytic function of myeloid cells, partial deficiency of complement proteins, and increased cytokine responses (21, 23, 63, 66, 68) (**Figure 1**). Secondary lymphoid compartments are also affected in DS, as patients exhibit abnormal proportions of peripheral blood lymphoid subsets (69–71), decreased function of natural killer (NK) cells (72), abnormal T cell development, and thymocyte maturation (15, 21, 22, 63, 73, 74) (**Figure 3**). Individuals with DS have a smaller thymus with reduced lymphocyte numbers (66, 75), which is thought to be the result of thymic dysfunction and apoptosis of B and T cells (68, 76). The thymus, even in newborns with DS, is smaller than that of infants without DS and exhibits structural abnormalities (17, 25), indicating that immunodeficiency and immune dysfunction are integral parts of the syndrome (17, 66). Below, we thoroughly detail the complex dysregulation of the various branches of the immune system in adults with DS.

### Dysregulation of Innate Immunity in DS

Individuals with DS exhibit significant alterations in the numbers and functionality of innate immune cells and innate immune receptors, all of which bear implications to susceptibility to infections. These alterations are described in detail in the following section.

#### Dysregulation of Innate Immune Cell Types in DS

##### Granulocytes

Granulocytes (neutrophils, eosinophils, and basophils) play a critical role in the first-line host defense mechanisms against a variety of microorganisms. In individuals with DS, neutrophils exhibit numerous abnormalities, including reduced chemotactic ability, associated with diminished resistance to infection (18, 77). Some adults with DS exhibit neutropenia (78) and a lower number of granulocytes (16).

Intracellular calcium ([Ca^2+^]i) acts as a second messenger in transmembrane signaling, regulates diverse functions in many cell types, and plays a role in neutrophil responses such as phagocytosis, locomotion, and free oxygen radical production (77). Yamato et al. reported that neutrophils in subjects with DS exhibit elevated levels of intracellular calcium concentration and prolonged Ca^2+^ response following neutrophils stimulation, suggesting an intrinsic neutrophil defect in DS (**Figure 3**).

Neutrophils from children with DS exhibit reduced chemotactic ability (79) and decreased CD11b expression, with a higher fold-change increase in CD11b expression following stimulation with LPS, compared with stimulated neutrophils from control subjects (66). These findings suggest that the ability of neutrophils to migrate and adhere is compromised in DS.

Mang and colleagues reported that 70% of the children with DS in their studied cohort show a significant degree of eosinopenia in the peripheral blood (80). Additionally, children with DS that suffer from hypothyroidism have eosinopenia in 38.5% of the cases, while pediatric hypothyroidism patients without DS exhibit eosinopenia in 11.8% of the cases (80). In neonates with DS, increased basophil number was reported (81). James and colleagues reported that both eosinophils and basophils from neonates with DS tend to be dysplastic with abnormal hypogranulation in 50% and 56% of the samples, respectively (82). Lastly, apoptosis of granulocyte was reported to be accelerated in DS under various conditions (41). Of note, most of the data on granulocytes in DS stems from studies in children, and more data is needed to assess this arm of the innate immune system in adults with DS.

##### Monocytes

Human monocytes are classified into classical CD14^++^CD16^-^, intermediate CD14^++^CD16^+^, and nonclassical CD14^+/dim^CD16^++^ cells (83). They display different chemokine-receptor expression profiles, potentially reflecting distinct tissue homing properties (84). Non-classical CD14^+/dim^CD16^++^ cells, which are elevated in DS (22), have superior antigen-presenting cell activity, they produce higher levels of proinflammatory cytokines (such as TNF and IL-10), and have a direct antibacterial activity in the tissue. In contrast, CD14^++^CD16^-^ monocytes, which are reduced in DS, exhibit a scavenging function and remove apoptotic neutrophils and debris (**Figure 3**). Children with DS were found to exhibit increased absolute numbers of CD14^+dim^CD16^++^ monocytes (16), which may contribute to chronic inflammation. Furthermore, monocytes from children with DS (age range: 6m-7y) exhibit a significant decrease in chemotaxis than monocytes from non-DS controls, which was not associated with age, sex, and physical development (85). Reduced monocyte chemotaxis was also reported by Barkin and colleagues, comparing institutionalized individuals with DS (mean age = 14.3) with non-DS institutionalized individuals (mean age = 13.9) (79).

Non-classical monocytes from children with DS express higher levels of TLR4 compared to controls. Following LPS stimulation, intermediate monocytes from children with DS exhibit an increase in the expression CD11b, which is involved in cell adhesion, compared with control subjects (66). These intermediate, inflammatory monocytes were also increased in adults with DS (22). Lastly, Kong and colleagues reported that monocytes from individuals with DS display elevated levels of Chr21-encoded IFN receptors and increased basal and stimulation-induced levels of pSTAT1 (86), suggesting hypersensitivity of trisomy-21 monocytes to IFN signals.

##### Natural Killer Cells

NK cells are found in higher frequencies in the peripheral blood of children with DS aged <2 years (16). This finding was also reported in adolescents (68) and adults, comparing individuals with DS to individuals with intellectual disability without DS (41). Nevertheless, NK activity, assessed by measuring the cytotoxicity of NK cells towards K562 target cells, was significantly reduced in DS compared with non-DS controls (87), which may contribute to higher susceptibility to viral infections in adults with DS (**Figure 3**). Waugh and colleagues assessed the expression of various markers associated with activation of NK cells and found that CD16, CD38, CD8, and CD11c were upregulated in CD56+CD16+ cytotoxic subset of NK cells from DS adults (22). This implies that the frequency of cytotoxic NK cells is not only elevated in DS but also exhibits an increased activation state. However, the functional cytotoxicity of these cells, which was previously reported to be reduced (87), was not measured in this specific subset.

#### Differences in Toll-Like Receptor Signaling

Toll-like receptors (TLR) are pattern recognition receptors (PRRs) that link between the activation of innate immunity and the initiation of adaptive responses. In mammals, the TLR family comprises 13 receptors, which can be grouped based on their subcellular localization: TLR1, TLR2, TLR4, TLR5, TLR6, and TLR10 are expressed on the cell surface, while TLR7, TLR8, and TLR9 are expressed in intracellular vesicles such as endosomes and lysosomes (88). TLR3 can be found both at the cell surface and intracellularly (89). TLRs can be activated by microbial-associated molecular patterns (MAMPs). For a detailed list of TLR exogenous ligands see Mukherjee (2016) (90). TLRs are also activated by endogenous, non-infectious molecules resulting from cellular injury, also referred to as damage-associated molecular patterns (DAMPs). Several TLR ligands of host cell origin have been identified, including but not limited to HMGB1 (High-mobility group B1), hyaluronan, β-defensin, heat shock proteins (HSPs), amyloids, and lipoproteins (91–94). For a more comprehensive list of putative endogenous ligands of TLRs see Erridge (2010) (95).

TLRs are amply expressed across immune cells, including neutrophils, macrophages, monocytes, lymphocytes, dendritic cells (DC), and microglia in the brain. Their signaling pathways lead to the induction of pro-inflammatory cytokines, chemokines, and co-stimulatory molecules, which are essential for initiating adaptive immune responses (96, 97). Pathological activation or dysregulation of TLR signaling can lead to an exacerbated production of inflammatory molecules and oxidative species, which has been associated with tissue damage, chronic inflammation, autoimmunity, and poorer outcomes during infections or acute sepsis (98); most of which are common in DS.

In fact, there is evidence that TLR signaling is altered in DS. Huggard and colleagues found that elevated TLR4 expression in LPS-stimulated non-classical monocytes increased neutrophilic response in pediatric patients with DS compared with controls (66). In addition, blood flow cytometry analysis showed that the basal expression of TLR2 is significantly higher in neutrophils and monocyte subsets in children with DS compared with controls (23). Given the large repertoire of DAMPs shown to activate TLR2, including anti-phospholipid autoantibodies, acute serum amyloid, HSPs, HMGB1, as well as several extracellular matrix components (95), some of which are known to be increased in DS serum (99), this elevation in TLR2 could potentially contribute to the state of exacerbated cytokine production characteristic of DS, *via* MyD88-independent pathways (100). This is compatible with additional findings in DS, indicating a significant reduction in the basal mRNA expression of MyD88, as well as no increase in its expression following stimulation with LPS (23). MyD88 is a key adaptor protein involved in the activation of TLR signaling, which is necessary for mounting immune responses to bacterial pneumococcal and streptococcal infections and antiviral responses (101). Therefore, its reduced expression could contribute to the greater susceptibility to such infections seen in DS.

Furthermore, dysregulation of MyD88-dependent TLR signaling in DS may be further exacerbated by mechanisms involving miRs, whose expression can be regulated by inflammatory cytokines. For example, the Chr21-encoded miR-155 represses the expression of MyD88 (102), which could further contribute to TLR dysregulation in DS (**Figure 3**). Tuttle and colleagues examined the detrimental effect of polyinosinic:polycytidylic acid (P(I:C)), a TLR3 agonist that triggers IFN responses, in the Dp16 mouse model of DS that recapitulates IFNRs triplication (103). The investigators reported that P(I:C) administration increased circulating levels of IFN-α in WT mice and Dp16, subsequently leading to weight loss and lethal immune hypersensitivity, specifically in Dp16 mice. In the lungs, P(I:C)-administered Dp16 mice exhibited elevated expression of IFNAR1, TLR3, and the key interferon-stimulated genes MX1 and EIF2AK2, compared with P(I:C)-administered WT mice, suggesting hypersensitivity to TLR3 stimulation in DS. This is specifically relevant for pathologies in which excess inflammatory responses correlate with disease severity, such as in the case of COVID-19 (104). Thus, TLR3 stimulation may lead to adverse IFN-mediated outcomes in DS. Importantly, Tuttle and colleagues were able to demonstrate that that P(I:C)-elicited weight loss and mortality can be overturned by JAK1 and JAK1/2 inhibitors, raising the possibility that these agents may be beneficial in individuals with DS suffering from autoimmune diseases (103).

Remdesivir, a broad-spectrum antiviral medication, was recently shown to shorten recovery time from COVID-19 (105). Moreover, recent findings suggest that combined administration of Remdesivir and Baricitinib, a JAK1/2 inhibitor, is superior to Remdesivir treatment alone. The combined treatment reduced the time of recovery from 8 days in Remdesivir-treated patients to 7 days, along with a 30% improvement in the clinical status. Moreover, patients that received non-invasive oxygen support at enrollment recovered at day 10 following Remdesivir/Baricitinib treatment, while Remdesivir-treated patients recovered at day 18. The 28-day mortality rate was also reduced from 7.8% in the control group to 5.1% in the combined treatment group (106). These mechanisms should be further explored in the context of infections in DS, as they could offer insights to uncover novel therapeutic avenues to correct the immune imbalance in people with DS.

Strict regulation of TLR pathways is crucial in protecting from infection but also in avoiding damage from excess cytokine production, a common phenomenon in DS, which can lead to worse outcomes, acutely in sepsis or chronic inflammation in autoimmunity.

#### Anomalies in FcγR Receptor Signaling

Fc receptors are a family of cell surface proteins that bind to the Fc portion of antibodies of a particular isotype (107). Cross-linking of Fc receptors and high affinity binding to Igs occur when these antibodies recognize pathogens or infected cells, stimulating phagocytosis of opsonized microbes by monocytes, macrophages, neutrophils, and DC or their destruction by cytotoxic effector cells (108). Activation of FcγRs constitutes an important function of the immune system in the removal of pathogens.

To date, a significant inhibition of FcγR was reported in individuals with DS aged 6 months to 32 years, due to an increase in FcγR blocking factors (109). Guarnotta and coworkers examined the sera of 29 individuals with DS and found that 55% of them displayed FcR inhibition above the upper limit of normality, compared with 7% in the control group. Moreover, the investigators found serum immune complexes, which may partially account for the observed FcR blockage, in a higher percentage of subjects with DS than in controls (109). Intriguingly, levels of FcγR1A and FcγR1B are significantly increased in a whole blood specimen, WBC, and monocytes of individuals with DS compared with non-trisomic individuals, as found using the TrisomExplorer database (http://www.trisome.org/explorer). A comprehensive study of the circulating proteome in DS revealed a significant downregulation of Fc-receptor Like 3 (FCRL3), an Fc-receptor-like glycoprotein involved in immune regulation (110). Several questions remain open, however, including whether FcγR signaling is compromised or exacerbated in DS, and of which FcR subtype(s) is affected, as well as the impact of such changes on immune activation and the ability to fight infections. From an immunotherapy perspective, there is a need to better understand FcR engagement, as this could influence the amount of antibody required to obtain a therapeutic effect or an adequate response to vaccines (111).

#### Interferon Hyperactivity

Four of the six interferon receptors (IFNR) are encoded on Chr21: the two subunits for type I IFN—IFNAR1 and IFNAR2; the type II IFNR subunit—IFNGR2; and a subunit of type III IFN—IL10RB (112, 113). Therefore, it is proposed that increased gene dosage of IFNRs results in consistent IFN response that contributes to the many clinical manifestations of DS (10). Sullivan and coworkers performed RNA-seq on fibroblast from individuals with and without trisomy-21 of different ages, genetic backgrounds, and genders. Their analysis revealed that the top upstream regulators that are predicted to be activated in DS are IFN-related factors. Indeed, they found a ~1.5-fold increase in the expression of the Chr21-IFNRs with relatively low inter-subject variability. Additionally, 21% of the Chr21 and non-Chr21 upregulated genes in individuals with trisomy-21 were linked to IFN signaling. Subsequently, the investigators reported that the IFN-activated kinases JAK1 and TYK2 are negative regulators of cell viability in trisomy-21 fibroblasts (10).

Consistent with their findings in fibroblast, Sullivan and colleagues found that all four IFNRs are upregulated in immortalized lymphoblastoids and that the strongest regulators of consistent gene expression are IFN-related factors. In circulating T cells and monocytes from donors with trisomy-21, IFNAR1, IFNAR2, and IL10RB were upregulated compared with controls. In association with this finding, T cells and monocytes were predicted to exhibit inactivation of the gene expression program driven by the N-Myc transcription factor and repression of the EIF2 pathway. IFN response is a selective control of protein translation that prevents the synthesis of viral protein following infection by impairing rRNA processing. Indeed, in monocytes and T cells, genes that overlap between the repressed MYCN and EIF2 programs encode components of the small and large ribosomal subunits, potentially impairing the integrity of protein synthesis in these cells (10).

In a substantial study, Waugh and colleagues performed deep mapping of the immune system of adults with trisomy-21 using mass cytometry to evaluate 100 cell types. Their analysis revealed a global immune dysregulation, including changes in lymphoid and myeloid cell compartments, associated with a widespread hypersensitivity to IFN-α. This could be explained by elevated expression of the type I IFNR subunit IFNAR1 across the entire immune system (22). Following stimulation with the type I IFN ligand IFN-α-2a, pSTAT1 and p4E-BP1 were significantly hyper-induced in multiple cell types tested from donors with DS, especially in myeloid lineage cells. pSTAT4 was hyper-activated in lineages involved in cellular immunity (T cells and CD7+ ILCs). CD8+ cells exhibited increased expression of Granzyme B (GZMB) following IFN-α-2a stimulation, and ERK1/2 phosphorylation was observed in monocytes and DCs from donors with DS (22). In line with these findings, Kong and colleagues reported an increased surface expression of the Chr21-encoded IFNAR1, IFNAR2, IFNGR2, and IL10RB, but not the Chr6-encoded IFNGR1 in EBV-transformed B cells from donors with DS (86). IFNAR2 and IFNGR2 were also overexpressed in monocytes from donors with DS compared to controls. Accordingly, levels of monocyte STAT1 and pSTAT1 were higher in DS than in controls but lower than in samples from donors with a gain of function STAT1 mutation, which enhances cellular responses to the three types of interferons. Indeed, following stimulation with IFN-α and IFN-γ, monocytes from individuals with DS exhibited elevated pSTAT1 expression (86). In a T cell-oriented study, Araya and coworkers demonstrated that trisomy-21 T cell subsets show elevated levels of basal IFN signaling and hypersensitivity to IFN-α stimulation that may contribute to increased autoimmunity (69). For example, pSTAT1 was hyper-activated in the CD4+ central memory T subset, and pSTAT4 peaked in CD8+ subsets. Without stimulation, CD8+ cells from donors with DS displayed increased activation marker IFN-γ expression compared with controls. CD8+ cells were also found to exist in an intermediate state, expressing both activation and inhibitory markers. For example, co-expression of IFN-γ and the inhibitory receptor PD-1 (69) can potentially reduce anti-viral responses, as high levels of PD-1 expression can have unfavorable immunological consequences during chronic viral infections (114).

As mentioned above, Tuttle and colleagues demonstrated that TLR3 agonist triggers an excessive type I IFN response that results in lethal immune hypersensitivity, specifically in Dp16 mice, in which IFNR subunits are triplicated (103). Furthermore, the investigators were able to overturn this effect using JAK1 and JAK1/2 inhibitors. Over-activation of type I IFN response by TLR3 stimulation is specifically relevant in acute conditions which involve excess inflammatory response, such as COVID-19 (104).

#### Dysregulation of the Complement Cascade

The complement system, a major effector mechanism of humoral and innate immunity, is composed of serum proteins, membrane-bound regulators, and receptors that promote a beneficial inflammatory response leading to pathogen opsonization and removal (115). Complement proteins are mainly produced by the liver; however, immune cells and endothelial cells can also contribute to the complement pool (116). Three complement pathways, activated by different recognition molecules, have been identified: the classical pathway, the alternative pathway, and the lectin pathway (115). They are characterized by sequential zymogen activation of distinct complement proteins. Although the initiator complement proteins in each pathway are distinct, all the pathways converge at C3 and C5 convertase enzyme complexes. C3 is cleaved into C3a and C3b, and C5 into C5a and C5b, of which C3b and C5b act as opsonins, and C3a and C5a as anaphylatoxins; the latter promoting the recruitment of phagocytes to the site of complement activation (115). In addition, deposition of C5b and other complement proteins (C6, C7, C8, and C9) results in the formation of a multimeric complex, termed membrane attack complex, which forms pores in the cell membrane and eventually promotes cytolysis (115). For more detailed reviews of the complement cascade see Merle (2015), Sarma (2011), and Ricklin (2010) (117–119).

**Figure 4:**
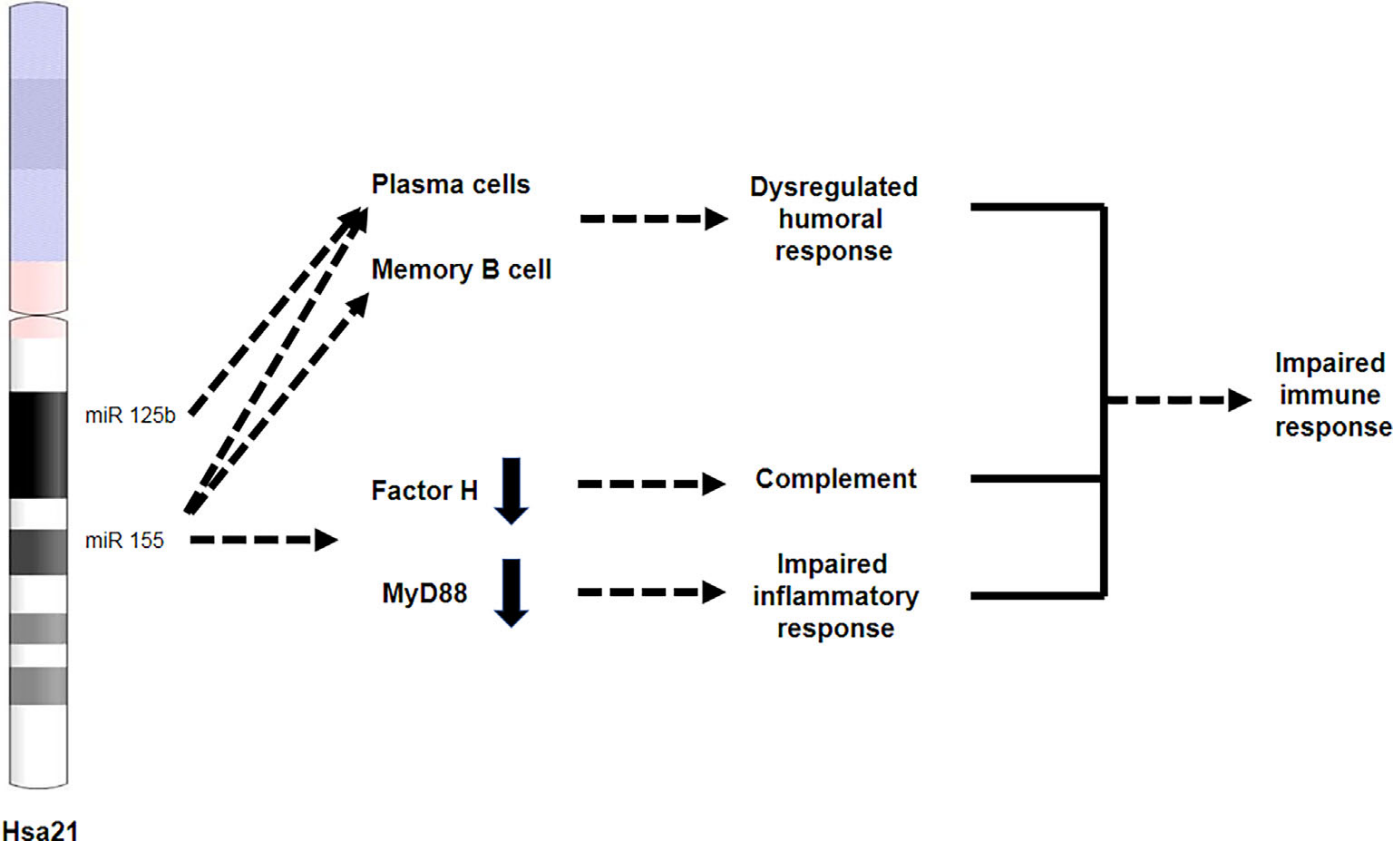
miR-125b and miR-155 are involved in immune dysregulation in DS. Both miR-125b and miR-155 are located on chromosome 21. miR-125b is overexpressed in plasma cells, while miR-155 is overexpressed in both plasma and memory B cells, leading to an impaired humoral response. miR-155 overexpression results in reduced expression of factor H and Myd88, leading to impaired complement response and impaired inflammatory response, respectively.

Activation of the complement in DS is implicated in DS-associated AD pathology (120, 121). It was mechanistically proposed that factor H, an inhibitor of complement activation, which is downregulated in brain, spleen, and liver tissues from older adults with DS (122), contributes to enhanced complement response in DS. Concordantly, the Chr21-encoded miR-155 (123), a negative regulator of factor H, was increased in the same tissue samples (122) (**Figure 4**). Therefore, these findings indicate that complement regulation may be compromised in DS, possibly resulting in abnormal complement activation in brain and peripheral tissues. Interestingly, recent studies further indicate a reduction in circulating complement proteins C3, C1Qa, C6, and C1R in the plasma of children and adolescents with DS (110). The authors proposed that this resulted from consumption of the complement system that is associated with excessive inflammation, and is particularly attributed to the increased IFN signaling characteristic of DS, and, as suggested, to other type I interferonopathies (110).

Dysregulation of the complement system, either by hyperactivation or by decreased complement levels, may result in a maladaptive inflammatory response (124), possibly contributing to the increased frequency of infections in DS, such as those related to the upper respiratory tract. Along this line, abnormal complement activation caused by the avian influenza virus H5N1 was shown to provoke acute lung injury, neutrophil infiltration into the lungs, and increased serum IL-6 and TNF-α levels in infected mice (125). Moreover, intravenous administration of either a C3aR antagonist or an anti-C5a antibody attenuated lung injury and neutrophil lung infiltration, diminished IL-6 and TNF-α levels, and increased the survival rate; thus, indicating that complement inhibition may be beneficial in influenza infections (125).

COVID-19 pathology is worsened by maladaptive immune response. It was therefore hypothesized that excessive complement activation contributes to disease progression and severity (126). Indeed, complement inhibition using antibodies directed against C5a (127), C5 (128, 129), and C3 (130) are currently being tested.

Sullivan and colleagues hypothesized that the dysregulated complement responses seen in DS could contribute to increased morbidity in this population (110). Indeed, complement consumption is associated with multiple pathologies, such as improper pulmonary clearance of *Streptococcus pneumoniae* (131), pathogenesis of age-related kidney injury (132), and microglia-mediated synapse loss in amyloid pathologies (133), which may further contribute to accelerating AD pathogenesis.

### Dysregulation of Adaptive Immunity in DS

#### Dysregulation of the T Cell Lineage

T lymphocytes exhibit a significant dysregulation in individuals with DS. In adults with DS, high levels of IFN-γ, which promotes Th1-responses and thus higher Th1/Th2 ratio, are thought to cause an imbalance between anti- and proinflammatory immune responses (68, 134). T cells from individuals with DS also express higher levels of IFN-stimulated genes because of heightened basal levels of IFN signaling and hypersensitivity to IFN-α stimulation (10, 69). CD8^+^ T cells from adults with DS are reduced in naïve subsets and enriched in differentiated subsets that express higher levels of activation and senescence markers (e.g., IFN-γ, Granzyme B, PD-1, KLRG1), and over-produce cytokines associated with autoimmunity (e.g., TNF-α) (69). Conventional CD4^+^ T cells display increased polarization toward the Th1 and Th1/17 states and produce higher levels of the autoimmunity-related cytokines IL-17A and IL-22. In concordance, levels of TNF-α, IL17A–D, and IL-22 are often upregulated in the plasma of people with DS. Additionally, regulatory, CD4^+^ and CD8^+^ T cells show higher expression of the inhibitory receptor PD-1, suggesting chronic antigenic stimulation and increased turnover of memory T cells in DS (68, 69).

Individuals with DS have a significantly higher number of unique T-cell receptor (TCR) gamma (TRG) sequences, along with decreased clonal expansion (72). TRG repertoire abnormalities may contribute to patients’ predisposition to infections and autoimmune diseases. However, since these observations were obtained using DNA sequencing of a mixed population of thymocytes, they cannot be specifically attributed to γδ T cells. Individuals with DS are reported to have a decreased population of cells expressing high levels of TCR αβ and approximately 10% of cells expressing TCR γδ (20, 135, 136) (**Figure 3**). The Chr21-encoded Ubiquitin-associated (UBA) and Src homology 3 (SH3) domain containing A (UBASH3A) is a negative regulator of NF-κB signaling in T cells that also play a broad role in autoimmunity (137, 138). UBASH3A is indeed upregulated in trisomy-21 T cells as found using the TrisomExplorer portal. Ge and collogues found that modulation of UBASH3A levels in unstimulated Jurkat T cells changes the amount of cellular CD3 chains and cell-surface TCR–CD3 complexes. Moreover, the investigators found that association between UBASH3A and components of cellular pathways plays a role in regulation of TCR–CD3 turnover and dynamics, including cell motility, endocytosis, and endocytic recycling of membrane receptors. Importantly, UBASH3A negatively regulated CD28 signaling and subsequently the activation state of T cells (137). It is therefore speculated that elevated T cell levels of UBASH3A in DS might impair T cell-mediated response to pathogens.

#### B Cell Dysfunction in DS

DS significantly affect both the differentiation and function of B cells, as fewer circulating B cells and impaired molecular maturation markers are consistently observed (17, 41, 139–141). Individuals with DS are sometimes B cell lymphopenic (23, 66, 73, 74) and exhibit a specific reduction in the number of switched memory B cells (17, 64) (**Figure 3**). The trisomy appears to alter the differentiation of B cells starting from pluripotent cells (75). In children with DS, all steps of peripheral B cell development are altered with a more severe defect during the later stages of B cell development. Transitional and mature-naïve B cell numbers are reduced by ~50%, as the bone marrow produces lower numbers of transitional B cells, while switched memory B cells are reduced to 10–15% of the numbers in age-matched controls (17). Moreover, the frequency of switched memory B cells specific to vaccine antigens is also reduced in individuals with DS compared with their euploid siblings (64). *In-vitro* studies revealed a rapid exhaustion of switched memory B cells in DS due to hypersensitivity to TLR9 stimulation-induced differentiation into antibody-producing cells (17, 64). Moreover, differentiation of T follicular helper (T_FH_) cells, which play an essential role in the formation of germinal center (GC) and the production of high-affinity antibodies, are skewed towards less effective CXCR3^+^ T_FH_ cells in children with DS (142). Type I IFN (IFN-I) plays a cardinal role in antibody isotype switching, and thus in the proper induction of humoral immunity following vaccines, as it activates DC and T_FH_ cells (143, 144). However, high levels of IFN-I can also impair antibody production in individuals with DS since it inhibits B cell encounter with viral antigens in draining lymph nodes (dLNs), interaction with T_FH_ and DCs, and subsequent activation, proliferation, and differentiating into antibody-secreting cells (145, 146). By restricting localization of T_FH_ cells within B cell areas of the spleen, IFN-I impairs GC formation, Ig-class switching, and plasmablast differentiation that subsequently results in suboptimal production of pathogen-specific IgM and IgG sub-classes, and resolution of infections (147). As such, immunoglobulin production in individuals with DS is impaired. Indeed, in addition to B cell lymphopenia, serum levels of immunoglobulin IgG2, IgG4, and IgM are decreased in children with DS, while total IgG, IgG1, IgG3, and IgA are increased in these individuals (**Figure 3**) (21, 23, 66, 73, 148). Additionally, IgA and IgG titers are largely reduced in saliva of adolescents and young adults with DS, although IgM levels appear to be normal (65). Thus, these reports indicate that the reduced number and rapid exhaustion of switched-memory B cells can impair the humoral response. As such, the B cell dysfunction appears to increase respiratory infections in young children with DS (21).

The prevalence of allergic sensitization is dramatically reduced in children with DS compared with children without DS (149). Indeed, Sullivan and coworkers reported that the levels of IgE antibodies, which mediate allergic responses (150), are reduced in individuals with DS (110). The investigators linked this observation to hypersensitivity to IFN-α/β in DS, which may suppress allergic inflammation by preventing activation of granulocyte and IL-4-mediated isotype switching to IgE (110).

#### Dendritic Cells in DS

Myeloid dendritic cells (mDCs), a heterogeneous population of professional antigen-presenting cells, serve as a bridge linking adaptive and innate immune responses (151). The absolute number of mDC is lower in pediatric patients with DS, while the number of plasmacytoid dendritic cells (pDC) is normal (16). In adults with DS, however, this reduction is not observed (22). mDCs also promote a Th1-type response of CD4^+^ T cells during viral infections by secreting a range of immunostimulatory cytokines and chemokines that prime differentiation of naïve T cells to effector T cells (151). Thus, Low mDC numbers in the peripheral blood may be associated with abnormalities in T cell maturation in DS (16) (**Figure 3**). Additionally, CD1c+ conventional dendritic cells, which have been proposed to promote autoimmunity through T cell activation, were observed in higher numbers in the circulation of adults with DS compared with age-matched controls (22).

pDCs, a rare subset of circulating DCs, also link innate and adaptive immunity, as they release high levels of type I IFNs following viral infections, which contribute to the activation of T cells and other pDCs (152). Indeed, pDCs from adults with DS overexpress IFNAR1, but they also display the largest fold change in STAT1 expression among all immune cells tested following type I IFN stimulation (22). Taken together, it is speculated that pDCs IFN-hypersensitivity in DS can contribute to severe outcomes of viral infections in which the course of the disease is influenced by excessive inflammation, as in the case of COVID-19. Indeed, plasmacytoid predendritic cells are rapidly diversified and activated to produce IFN-α and other cytokines following interaction with SARS-CoV-2 viral strains (153).

Overall, a selective cell-mediated immunodeficiency, defective neutrophil chemotaxis, low T and B cell counts, and impaired antibody response to pathogens presumably explain the higher rate of mortality from pneumonia and other respiratory diseases in children with DS (21, 65).

### Aberrant Cytokine and Chemokine Signaling in DS

Cytokines participate in the regulation of immune and inflammatory responses through binding to specific receptors and activation of signaling pathways; many of them acting through the Janus kinases (JAKs)/signal transducer and activator of transcription proteins (STAT) pathways (154). The secretion of cytokines is a normal part of a cell’s physiological response to infection and pathogen recognition; however, in certain pathological contexts, an exacerbated production or an improper resolution can lead to sustained inflammation and associated chronic damage (107). In the context of respiratory infections, which are prevalent in individuals with DS, inflammatory cytokines have been associated with poorer prognosis or fatal outcomes (155). Likewise, elevations in IL-6, TNF-α, macrophage inflammatory protein 2 (MIP-2), and the IL-1 receptor antagonist (IL-1RA) have been associated with increased mortality in a murine model of septic shock (156).

Individuals with DS exhibit higher circulating levels of pro-inflammatory cytokines and chemokines than euploid controls; even in the absence of infections or autoimmunity (69, 110, 157–161). A meta-analysis that reviewed 19 studies that included almost 1500 participants concluded that circulating IFN-γ, IL-1β, TNF-α, and MIP-1α are significantly elevated in individuals with DS (157, 162). Moreover, a proteomic analysis of the plasma and serum from children and adults with DS revealed a significant elevation in the pro-inflammatory cytokines IL-6, MCP-1, IL-22, and TNF-α (110). The circulating cytokine signature resembles that of type I IFN pathologies and several autoimmune conditions, many of which are indeed common in DS (163, 164).

These heightened basal levels of inflammatory cytokines seen in DS across multiple age brackets can be in part attributed to the trisomy-21, but can be also related to the fact that DS is characterized by priming of diverse immune cell types, reflecting heightened pro-inflammatory states (69). This has been recently demonstrated in a comprehensive study characterizing the peripheral T cell compartment in adults with DS, where it was shown that CD8^+^ T cells from participants with DS stimulated *in vitro* responded more potently than their euploid counterparts, overproducing TNF-α, IFN-γ, IL-2, and MIP-1α, MIP-1β, Eotaxin, GM-CSF, IL-8, IL1-RA, and IL-10 (69). Similarly, CD4^+^ T cells from people with DS stimulated *in vitro* also expressed higher levels of IL-10, IL-17A, IL-22, and MIP-3α, which is consistent with a polarization of these cells toward the Th1 and Th17 states. Moreover, this study also showed that effector T cells in DS are resistant to CD4^+^ Treg mediated suppression, bringing additional evidence to explain the chronic heightened inflammatory state characteristic of DS.

The consequences of systemic exacerbated inflammation are of particular concern in the context of viral respiratory infections because of the link between cytokine storm, disease severity, and mortality (165). Indeed, Broers and colleagues reported that *ex vivo* stimulation of whole blood from children with DS with influenza-A virus resulted in increased levels of TNF-α, IL-1β, IL-6, IL-8, and IFN-α compared to their euploid siblings (166). In addition, elevated levels of IL-10, an IFN-induced cytokine, may further explain the high susceptibility of people with DS to develop pneumococcal pneumonia following viral infections (6, 167). This is since this anti-inflammatory cytokine may reduce macrophage and neutrophil function, compromising the body’s anti-bacterial defense (168). Therefore, upon viral infections, individuals with DS should be considered for prophylactic antibiotics such as azithromycin (169). Lastly, it has been recently speculated that higher basal and stimulation-induced cytokine release from trisomy-21 immune cells contribute to poorer outcomes following SARS-CoV-2 infection in patients with DS (104). COVID-19 morbidity and mortality are indeed driven by an exacerbated immune response that may result in a cytokine storm and subsequent organ failure. As mentioned above, a combined treatment with the JAK1/2 inhibitor Baricitinib, and Remdesivir in COVID-19 patients promotes recovery and reduces COVID-19 mortality rates compared to Remdesivir treatment alone (106). Additionally, in a phase-3, global, double-blind, randomized, placebo-controlled trial by Marconi and colleagues, COVID-19 patients received the corticosteroid dexamethasone, in combination with Baricitinib or placebo. The investigators reported that although reduced disease progression did not achieve statistical significance, mortality rate was reduced by 38.2% in patients that received the combined treatment (170). This promising path could be further examined in clinical trials that include individuals with DS, as JAK1/2 inhibition may be even more beneficial in COVID-19 patients with DS.

### Implications for Vaccine Efficacy in DS

Immune dysregulation appears to be a major obstacle in generating protective immunity through vaccination (171). Elderly individuals (172), people with diabetes (173), morbidly obese individuals (174), and individuals with DS (57, 73, 175) are considered to poorly respond to vaccines. Due to suboptimal primary and memory immune responses, vaccines often inefficiently protect people with DS against several pathogens. Pneumococcal capsular polysaccharide vaccine (21) elicits a suboptimal response in children and adolescents with DS, which can be reversed using a conjugated pneumococcal vaccine, which induces a T-dependent response (73). Following a Hepatitis B virus (HBV) vaccination, only 31.9% of children with DS after the age of 10 exhibited adequate anti-HBV titers (25). No difference in seroconversion was found between individuals with DS and controls following a Hepatitis A virus vaccination (176). Individuals with DS also show a decreased avidity of the antibody response to tetanus toxoid booster vaccination at 9 years of age, suggesting impaired memory B cell selection in the germinal center (175). Response to influenza virus A and B vaccinations seems to be adequate in patients with DS despite the decreased number of CD19^+^ B cells (74). Other studies found no impaired vaccine responses in individuals with DS (between 2 and 18 years old) to T-independent and -dependent vaccines, as both type-2 pneumococcal polysaccharide and inactivated seasonal influenza vaccines elicited a good humoral response. In contrast, influenza and pneumococcal glyco-conjugated vaccines induced less IgM in the saliva of children with DS of ages 6-7 than in controls, despite comparable production of IgG and that of IgA (64). On the other hand, the deficiency in vaccine-induced protection can be circumvented by infusion of a pathogen-specific antibody. As previously mentioned, administration of Palivizumab, an RSV neutralizing monoclonal antibody, reduces RSV-related hospitalization in children with DS (<24 months old) (55). In sum, the efficacy of some traditional vaccine strategies may be lessened in individuals with DS, underscoring the need for more research in this area to enable novel and DS-tailored strategies. To date, no data is available regarding the efficacy of the various COVID-19 vaccines in individuals with DS in comparison to the general population.

## Genetic Basis for Immune Dysregulation and Increased Burden of Infections in DS

Multiple genes within the triplicated Chr21 are directly associated with the immune response and regulation and may contribute to immune dysregulation in DS (**Figure 2**). As detailed in previous sections, four of the six IFNR are encoded on Chr21 and are upregulated in virtually all immune cells, causing a variety of immune dysregulations related to IFN hypersensitivity (10, 22, 69, 86, 103). Clinically, it is speculated that consistent IFN-response and IFN hypersensitivity may worsen the outcomes of respiratory tract infections.

β-2 integrin is encoded by the Chr21 ITGB2 gene. Upon association with CD11a, ITGB2 forms the LFA-1 protein (integrin αLβ2), which is implicated leukocyte migration, T cell differentiation, and neutrophil arrest (177, 178). In association with CD11b, ITGB2 forms the MAC-1 protein (integrin αMβ2), which is implicated in internalization of bacteria to phagocytes (179), and macrophage fusion (180), but also serves as complement receptor 3 (CR3) (181). Upon association with CD11c, ITGB2 forms the complement receptor 4 (CR4, integrin αXβ2) (181). ITGB2 association with CD11d forms integrin αGβ2, which mediates NK-neutrophils interaction and macrophage fusion, retention, and migration (180, 182). ITGB2, which is critical for leukocyte migration, is over-expressed in both CD4^+^ and CD8^+^ T cells from peripheral blood of children with DS (11). Paradoxically, overexpression of ITGB2 in lymphocytes results in poor adherence *in vitro* (183). Indeed, ITGB2 is upregulated in the whole blood, WBCs, monocytes, and T cells in individuals with DS, as indicated in the TrisomExplorer database. This can potentially explain the chemotaxis deficit seen in some DS immune cells, such as neutrophils and monocytes (79, 85).

ICOSLG (also known as B7 or CD275) encoded on Chr21 is involved in Treg cell function (22, 183, 184). ICOSLG is also constitutively expressed in human monocytes and dendritic cells, and its expression is upregulated in monocytes by IFN-γ (185). Indeed, according to the TrisomExplorer database, ICOSLG is upregulated in trisomy-21 monocytes.

AIRE, a protein whose gene is also located on Chr21 and is known to play a critical step in preventing autoimmunity by regulating the apoptosis of cells expressing TCR against self-antigens, is downregulated in DS patients, according to some reports (13, 186). However, according to the TrisomExplorer database, AIRE expression remains unchanged in whole blood, WBCs, and monocytes, and is upregulated in T cells from individuals with DS.

UBASH3A, a negative regulator of NF-κB signaling in T cell (137, 138), is upregulated in trisomy-21 T cells as found using the TrisomExplorer database. It can be speculated, therefore, that elevated T cell levels of UBASH3A in DS may impair T cell-mediated response to pathogens.

Regulator of Calcineurin 1 (RCAN1), also known as DS critical region 1 (DSCR1), is a Chr21 gene that encodes a protein that inhibits the Ser/Thr phosphatase calcineurin (187). Using a mouse model, Martin and colleagues were able to demonstrate that RCAN1 overexpression causes T cell abnormalities, as described in DS (188). These changes include T cell developmental defects, reduced number of mature CD4+ and CD8+ thymocytes, reduced T cell number in immune organs, reduced proliferative capacity, and aberrant cytokine production by T cells.

Runt-related transcription factor 1 (RUNX1), a Chr21-encoded transcription factor of the runt domain-containing family, is a regulatory factor in T-cell immunity through interaction with multiple master regulators involving the differentiation and function of T cells (189). Overexpression of RUNX1 was associated with thrombocytopenia, possibly through regulation of Th17 cell differentiation (190). Moreover, overexpression of the Runx1 transcription factor impairs the development of thymocytes from the double-negative to double-positive stages (191). Therefore, RUNX1 triplication is a potential mechanism that contributes to T cell dysregulation in DS. Additionally, targeting of RUNX1 by CD82 is essential for macrophage phagosome arrest, required for the intracellular growth of *Mycobacterium tuberculosis* (MBT), as knockdown or knockout of CD82 or RUNX1 increased antibacterial host defense (192). Thus, we speculate that under MBT infection, RUNX1 overexpression, as occurs in WBCs in DS according to the TrisomExplorer database, may reduce macrophage response.

However, only some of these genes were found to be overexpressed in subjects with DS under physiological conditions. For example, Martinez et al. reported that ITGB2, IFNAR1, IFNGR2, and B7 are not over-produced at the protein level in T cells, B cells, monocytes, and neutrophils from patients with DS (183); however, immune stimulation can alter their protein expression.

Besides changes in gene expression, several other genetic mechanisms of immune dysregulation in DS have been proposed. Farroni and colleagues linked miR-155 and miR-125b, encoded on Chr21, to B cell defects in DS (193). Additionally, the expression of miR-155 and miR-125b in memory B cells and miR-125b in plasma cells is increased. *In-vitro* inhibition of miR-155 changed the fate of B cells, partially correcting the B cell defects observed in DS (**Figure 4**).

From an epigenetic perspective, Kerkel et al. found gene-specific abnormalities of CpG methylation in peripheral blood leukocytes and T cells in adults with DS (194). Many of the differentially methylated genes are known to regulate lymphocyte development and function, such as Transmembrane Protein 131 (TMEM131), Transcription Factor 7 (TCF7), CD247, SH3 Domain Binding Protein 2 (SH3BP2), Eukaryotic Translation Initiation Factor 4E (EIF4E), Phospholipase D Family Member 6 (PLD6), Small Ubiquitin Like Modifier 3 (SUMO3), Nucleotide Binding Oligomerization Domain Containing 2 (NOD2), and Carnitine Palmitoyltransferase 1B (CPT1B) (194).

## The Immunosenescent Phenotype of DS

Although individuals with DS have significantly longer lifespan nowadays, they often show early signs of aging-related disorders (40, 46, 195) as well premature aging of multiple body tissues (196).

Telomeres are chromosome ends consisting of highly conserved TTAGGG repeats that progressively shorten with age (197). Short-term T lymphocyte cultures from people with DS and AD-related dementia have shorter telomeres than T-lymphocytes from age- and sex-matched people with DS with no dementia (198, 199). This phenomenon precedes late stages of dementia in DS, as shorter telomere length was also measured in adults with DS and mild cognitive impairment (MCI) compared to age- and sex-matched individuals with DS and without MCI (200).

Due to the presence of multiple immune-related genes within Chr21, it is a complex feat to differentiate between developmental and aging-related impairments in the immune system of individuals with DS. Changes in the immune system of individuals with DS may occur due to precocious aging or early immune deficits followed by normal aging, though some lines of evidence suggest it is not precocious immunosenescence (201). Therefore, the mechanisms involved in age-related decreased function of the immune system in individuals with DS are complex and not well defined (24, 196).

A smaller thymus, as measured by thymus-thoracic ratio, can be identified in fetuses with DS, which reflects thymic involution and not hypoplasia (202–205). Infants with DS have small thymuses with fewer lymphocytes, smaller thymic cortex, and lack corticomedullary borders that resemble thymic involution (19, 205). With age, the thymus is disposed to involute, and fewer cells needed for adaptive immunity are produced, resulting in a reduced repertoire of B and T cells in elderly individuals in general, and especially those with DS (201).

DNA methylation patterns generate an “epigenetic clock”, which correlates to chronological age (206, 207). Using this paradigm, Mendioroz and coworkers reported accelerated aging of CpG methylation patterns in DS during fetal and possibly early post-natal development, but not during adulthood (208). In fact, DNA methylation in T cells is elevated in individuals with DS and is increasing at the same rate as in euploid individuals. Younger individuals with DS, however, start at a higher level of DNA methylation and display early epigenetic aging rather than accelerated epigenetic aging (209). This remarkable finding establishes that early epigenetic aging, but not accelerated epigenetic aging, occurs in DS. Kerkel and colleagues conducted DNA methylation profiling in peripheral blood leukocytes from trisomy-21 subjects. They reported gains and losses of DNA methylation that strongly affect approximately 100 genes that are uniformly distributed across chromosomes (194). Interestingly, these methylation differences are not associated with age effects, as fewer than 5% differentially methylated CpG loci in DS show evidence of age-dependent methylation (208, 209).

Some immune alterations in DS are age-dependent: a decrease in absolute numbers of T lymphocytes (CD3^+^), involving both CD4^+^ and CD8^+^ subsets, increased number of activated T cells (CD3^+^, HLA-DR^+^), marked decreased numbers of B lymphocytes (CD19^+^), increase in some subclasses of Ig, and increase in the number of cells with markers of NK activity (41) (**Figure 3**). This is accompanied by suboptimal antibody responses to immunization (23, 66), as the initial response to vaccines is generally adequate in individuals with DS but shows lower mean titers and a need for more frequent booster immunizations compared to individuals without DS (25). Many of these immunological alterations are age-related and can be enclosed in the spectrum of multiple signs of early senescence of the immune system (41, 63). For example, alterations in adaptive immunity, lack of diversity among naive cells, and altered development of humoral immune responses after vaccinations are all reminiscent of immuno-senescence typically seen in the elderly (41, 68).

Individuals with DS are subjected to global immune dysregulation and chronic inflammation (22), which is also implicated in aging (210, 211). Therefore, early immunosenescence seen in DS may be linked to life-long immune hyperactivity. Age-related decline in immunity is characterized by stem cell exhaustion, telomere shortening, and disruption of intercellular communication (212). Indeed, chronic inflammation is closely linked to these molecular and cellular deficits. DS co-morbidities such as cardiovascular diseases, pulmonary diseases, and autoimmune conditions can also contribute to telomere shortening and thus to senescence (212). Steady-state differentiation of hematopoietic stem and progenitor cells into myeloid lineage cells is controlled by growth factors, such as G-CSF, M-CSF, GM-CSF, and Flt3-L. Levels of these factors may be modified during chronic inflammation by cytokines such as IFN-γ (212). Additionally, inflammation-induced DAMPs may result in excessive TLR activation in immune cells, resulting in accelerated cellular aging (212). Mitochondrial ROS production is increased in DS (213) and thus may contribute to immunosenescence *via* a range of different mechanisms (214).

In sum, changes in the immune system in individuals with DS, compared to individuals without DS occur at early stages and continue to affect individuals at an increased rate throughout their lifetime, by mechanisms of telomere shortening, earlier epigenetic aging, chronic inflammation, and immune hyperactivity.

## Concluding Remarks

Despite the high prevalence of DS in the population, relatively little is done to understand and protect adults with DS from infections, including current and future pandemics. Analysis of the immunological, epidemiological, and clinical landscape in DS reveals the need for DS-tailored protective and therapeutic strategies. Because of unique immune impairments and heightened inflammatory responses, people with DS display a suboptimal response to vaccines; they are more susceptible to infections of the respiratory tract and have higher hospitalization rates, complications, and mortality worldwide.

## Author Contributions

TI, ABi, MFI, LF-A, MD, IDT, SEA, EY, YH, M-CP, ABo, RR, BS, JL, WM, AS and EO wrote the manuscript. TI and EO prepared the final version. All authors contributed to the article and approved the submitted version.

## Conflict of Interest

The authors declare that the research was conducted in the absence of any commercial or financial relationships that could be construed as a potential conflict of interest.
